# Action over anthropometrics: an action-scaled framework for youth sports modifications

**DOI:** 10.3389/fpsyg.2025.1559656

**Published:** 2025-03-18

**Authors:** Ran Zheng, John van der Kamp

**Affiliations:** Department of Human Movement Sciences, Faculty of Behavioural and Movement, VU Amsterdam, Amsterdam, Netherlands

**Keywords:** modified sport, body-scaling, action-scaling, gender, collective behavior

## Introduction

In junior sports, equipment, pitch dimensions, and team sizes are typically modified to better suit the action system of younger athletes (for a review, see Buszard et al., 2016). These modifications are aimed to attain the same dynamics and task constraints in youth games as in adult games (Limpens et al., 2018). For example, the Dutch Football Association (KNVB) recommends that children under 10 years of age play soccer in a 6 v 6 format on a reduced pitch measuring 42.5 by 30 meters, about half the size of an adult pitch, and with goals sized at 5 by 2 meters. According to sports organizations like FIFA, these adjustments benefit both stakeholders and players. For stakeholders, smaller pitches are more economical to build and maintain, while for players, the smaller format encourages frequent ball touches and greater involvement, enhancing enjoyment, skill development, and overall engagement with the sport (FIFA, 2013). As a result, sports programs increasingly use these modifications to support participation and talent development (Buszard et al., 2022).

However, the guidelines for (re-)designing youth sport environments remain unclear and often are arbitrary (Buszard et al., 2016), raising significant issues. For instance, the absence of a conceptually coherent and standardized framework for these modifications has created inconsistencies, with sports organizations varying in their guidelines and frequently updating their standards (FIFA, 2013). To address these issues, Broadbent et al. (2021) recently proposed a body-scaled approach for sports modification, suggesting that adjustments to pitches or equipment should reflect the anthropometric relationship between children and adults (see also Limpens et al., 2018; Azmi et al., 2024; for a review, see Buszard et al., 2016). In this opinion paper, we first discuss the advantages and limitations of the body-scaled approach in youth sports modifications and then propose an action-scaled approach. Additionally, we highlight two often-overlooked aspects of youth sports that are also brought into focus by the action-scaled approach and warrant further attention.

## Body-scaled vs. action-scaled framework

### Body-scaled approach

The concept of body-scaling was first introduced by Warren (1984) in his seminal study on stair climbing. He investigated how people's perception of whether stairs were climbable was related to their body measurements. Warren observed considerable variation among young adults in the step heights they perceived as climbable. However, he found that, regardless of individual differences in body height, stairs were consistently perceived as climbable when the step height was < 0.88 times the individual's leg length. When step heights exceeded this ratio, stairs were perceived as unclimbable. From this, Warren concluded that individuals perceive affordances (i.e., possibilities for action, Gibson, 1979) by scaling environmental properties to their body measures. Since then, this body-scaled approach has been validated across various tasks, including sitting (Mark et al., 1990), reaching and grasping (van der Kamp et al., 1998), and walking through narrow openings (Warren and Whang, 1987).

Building on this foundation, the body-scaled approach has recently been applied to youth sports, where researchers and practitioners adapt equipment and field dimensions to fit young athletes' body measurements (e.g., Limpens et al., 2018; Gorman et al., 2021; Broadbent et al., 2021; Azmi et al., 2024). These modifications aim to maintain similar proportions or ratios between the size of equipment or fields' and players' body measurements, replicating the ratios typically found in adult sports (Limpens et al., 2018). Research has shown that such adjustments benefit young players by improving accuracy and technique (Timmerman et al., 2015) and encouraging greater functional variability (Buszard et al., 2020). Furthermore, these changes enhance young players' self-efficacy, leading to more positive and enjoyable experiences in sports (Gimenez-Egido et al., 2023).

### Action-scaled approach

Despite its widespread popularity and application, the body-scaled framework has not been without its critics. One of the earliest critiques came from Konczak et al. (1992), who noted that stair climbing involves two key tasks: first, placing one foot on an elevated surface, and second, shifting the center of mass over the newly positioned foot to establish it as the new base of support while extending the weight-bearing leg. They argued that additional biomechanical factors, such as muscle strength and joint flexibility, could influence the perception of climbability beyond leg length alone. Their experimental findings supported this argument, showing that both perceived and actual climbing ability among older adults were influenced not only by body anthropometrics but also by muscle strength and joint flexibility.

These findings underscore a key limitation of the body-scaled approach: affordance perception is not solely determined by body dimensions. Instead, it is shaped by a broader range of factors, including coordination, agility, and strength, which evolve throughout development. This insight has significant implications for fields such as youth sports, where these additional factors play a crucial role in shaping how individuals perceive affordances. Rooted in ecological psychology, affordances reflect the relationship between an individual's action capabilities and relevant environmental properties (Gibson, 1979; Fajen, 2007). Therefore, action capabilities are a multidimensional concept, influenced by a range of human characteristics, such as body anthropometrics, coordination, agility, and strength. However, they cannot be fully captured or defined by any single factor.

Movement control is guided by the perception of affordances, with performers moving in ways that sustain affordance perception (Fajen, 2007). As a result, variations in action capabilities among individuals lead to different perceived possibilities for action (Vauclin et al., 2024). For instance, faster goalkeepers may perceive some penalties as stoppable, while slower goalkeepers may not (Zheng et al., 2021, 2022). Similarly, modifications to environmental properties—such as adjusting equipment or pitch dimensions—can create new affordances. For example, reducing the ball's weight enables children to shoot more accurately and pass farther than they could with a standard-weight ball during a basketball game (Arias et al., 2012).

Building on this understanding, while modifications based on the body-scaled approach simplify the task, they do not maintain the same dynamics as the adult game. This is because body measurements alone may not fully account for a performer's action capabilities (Konczak et al., 1992). Although younger players often demonstrate improved performance in body-scaled sports settings compared to unmodified ones, these skills may develop exclusively within the context of the body-scaled game and not transfer effectively to adult games. To address this limitation, scaling based on task-specific action capabilities is recommended. This approach considers the ratio between the task-specific action capabilities of children at different developmental stages and those of adults, as expressed in the following Equation 1:


(1) $$\begin{array}{l} \frac{\text{Task constraints for adults}}{\text{Task}-\text{specific action capabilities of adults}}\;=\\ \;\frac{\text{Task constraints for youth}}{\text{Task}-\text{specific action capabilities for youth}} \end{array}$$

For instance, in tennis, the primary task-specific action capability is ball-striking speed, which directly influences time pressure (Buszard, 2022). If the ball speed in adult games is twice as fast as in U10 children's games, the length of the modified court for U10 children should be half that of the adult court to maintain comparable task demands. By aligning task constraints with players' action capabilities, the action-scaled approach creates an environment for youth that offers a similar set of affordances as the adult game. This alignment enhances skill development and increases the likelihood of a smoother transition from children's games to adult-level performance.

## Gender differences

A significant yet often overlooked aspect of modifying youth sport programs is the consideration of gender differences. Research has traditionally focused on male youth athletes, leaving female participants underrepresented. This creates a knowledge gap in understanding how gender shapes the design of enriching sports and learning environments. Before puberty, boys and girls typically exhibit similar anthropometric characteristics and action abilities (e.g., De Ste Croix et al., 2002; Broadbent et al., 2021). However, as they mature, their developmental trajectories diverge. Adult men generally become larger, faster, and stronger than women, leading to notable differences in maximum action capabilities (Broadbent et al., 2021). Consequently, the same environment can offer different affordances to men and women.

For example, Zheng et al. (2022) compared the penalty goalkeeping behaviors of adult male and female goalkeepers that compete at similar skill levels. Female goalkeepers required more time to defend a regular sized goal. Additionally, female kickers produced less powerful shots, resulting in longer ball flight times when kicking the ball from the regular distanced penalty spot. Together, these factors create stricter constraints for female goalkeepers, as their action capabilities differ more markedly from men than those of the kickers. Consequently, the possibilities for action vary between male and female goalkeepers during a soccer penalty kick. Specifically, female goalkeepers are constrained to start their dives early and thus are more susceptible to deceptive actions of the kicker compared to their male counterparts (Zheng et al., 2022).

Gender differences in action capabilities underscore the need for thoughtful design in modified sports. Before puberty, using identical equipment and playing fields for boys and girls may result in undersized setups for girls (Broadbent et al., 2021). After puberty, as boys grow faster and stronger, these same fields and equipment may become oversized for girls, making tasks more challenging and potentially reducing their engagement. This challenge is evident in reports of many secondary school-aged girls (12–18 years old) avoiding physical education classes due to feelings of incompetence (Hortigüela-Alcalá et al., 2021). Addressing these issues requires accounting for gender differences in action capabilities when designing youth sports guidelines. The action-scaled approach can ensure sports environments are tailored to the distinct action capabilities of boys and girls, promoting inclusivity and engagement.

## Collective behavior

Most research on modified sports has concentrated on individual sports (e.g., Limpens et al., 2018) or specific technical skills within team sports (e.g., Arias et al., 2012). However, the influence of modified sports on the collective behavior of young players remains largely unexplored. From an ecological psychology perspective, collective behavior in sports is guided by shared team affordances, or the opportunities for action perceived collectively by a group of players (Silva et al., 2013). Perceiving these shared affordances requires players to perceive not only their own maximum action capabilities but also that of their teammates and opponents (Silva et al., 2013). Consequently, action capabilities play a more important role than body anthropometrics in perceiving shared affordances. The action-scaled approach is thus more suitable than the body-scaled approach for supporting the development of shared affordances in modified sports.

The primary task constraint influencing the perception of shared team affordances is the distance between players, which is shaped by sport modifications such as pitch dimensions and player numbers (Clemente et al., 2020). This distance is closely tied to players' maximum action capabilities, such as how far they can kick the ball or how quickly they can intercept it (Travassos et al., 2012). Smaller pitches with more players reduce player distances, limiting exploration and decreasing variability in collective behaviors (Olthof et al., 2018). Additionally, player distances influence the attunement to shared team affordances: shorter distances encourage verbal communication, while longer distances promote reliance on non-verbal communication, such as eye contact (Caso and van der Kamp, under review).^1^

These shifts in communication indicate that players adapt to different information sources as distances change, resulting in distinct patterns of collective behavior.

Building on the role of action capabilities and player distances in shaping shared team affordances, we propose the action-scaled approach as a framework to support the development of collective behavior in modified youth sports. This approach aims to provide youth players with a set of affordances comparable to those in adult games, ensuring that representative team affordances are effectively integrated into their environment. However, further research is needed to identify the specific shared affordances and the underlying team-action capabilities in which they are grounded.

## Conclusion

This opinion paper evaluated the strength and limitations of the body-scaled approach in youth sports modifications and advocates for the action-scaled approach as a more effective alternative. By aligning task constraints with players' action capabilities, the action-scaled approach ensures that the collection of affordances available closely mirror those in adult games, fostering skill development that transitions smoothly to adult performance. Additionally, the paper highlights the importance of addressing gender differences and collective player behavior, filling critical gaps in current practices and paving the way for more inclusive and effective youth sports frameworks. For practical implementation, sports organizations should collaborate with sport scientists and experienced practitioners to identify the task-specific action capabilities required in each sport. They should then assess how current youth modifications align with adult games using the action-scaled approach. For example, they should determine whether these modifications are oversized or undersized and adjust them accordingly. Furthermore, future research should empirically evaluate the effects of these action-scaled modifications on youth performance across different sports and developmental stages. Such studies would offer essential evidence to support the broader adoption of this innovative framework and further enhance its application in youth sports settings.

## Footnotes

^1^Caso, S., and van der Kamp, J. (under review). An ecological perspective on intra-team communication in elite football. *Int. Rev. Sport Exerc. Psychol*.
